# How to manage surgical patients during and early after COVID-19 pandemic: a practical approach for low-and middle-income countries (LMICs)

**DOI:** 10.11604/pamj.supp.2020.35.2.23681

**Published:** 2020-06-09

**Authors:** William Ngatchou, Mazou Ngou Temgoua, Joel Noutakdie Tochie, Fabrice Stephane Arroye Betou, Jean Cyr Yombi, Daniel Lemogoum

**Affiliations:** 1 Department of Surgery, Douala Faculty of Medicine and Pharmaceutical Sciences, University of Douala; 2 Department of Internal Medicine and sub-Specialities, Faculty of Medicine and Biomedical Sciences, Yaoundé, Cameroon; 3 Departement of Cardiovascular and thoracic surgery, University teaching Hospital of Fann, Cheikh Anta Diop University, Dakar, Senegal; 4 Department of Anaesthaesiology and Critical Care Medicine, Faculty of Medicine and Biomedical Sciences, Yaoundé, Cameroon; 5 Department of Infectious Diseases and perioperative medicine, Saint Luc University Hospital, Université Catholique de Louvain, Brussels, Belgium; 6 Hypertension clinic, Department of cardiology, UBL-Erasme Hospital, Free Brussels University, Brussels, Brussels, Belgium; 7 Department of Medicine and specialties, Faculty of Medicine and Pharmaceutical Sciences, University of Douala, Douala, Cameroon

**Keywords:** Surgery, COVID-19, low-and middle-income countries

## To the editors of the Pan African Medical Journal

Coronavirus disease 19 (COVID-19) pandemic caused by severe acute respiratory syndrome (SARS-CoV-2) is a global public health problem with major economic and social consequence of tremendous importance. Since the identification of its first case in Wuhan, Hubei, China in end December 2019, the exponential and rapid spread of the disease over all continents led the World Health Organization (WHO) to declare a pandemic in March, 11, 2020. Since, then several governement and non-governmental organizations pay a serious attention on this pandemic [1]. Moreover, the rapid spread of the pandemic is very challenging for its control by placing enormous pressure on all health care systems worldwide [2], and on frontline healthcare workers who are rapidly altering their professional responsibilities to help meet hospital needs. Limitation of staff members, redeployment of healthcare providers in the COVID-19 units, priority allocation of material and financial resources to fight COVID-19, limitation of the number of outpatient consultations have all contributed to a global health economic crisis [3]. It is estimated that health care workers (HCW) comprise between 4% and 19% of all reported COVID-19 cases in Europe and China [4]. Their risk of being contaminated is particulary high when treating non diagnose COVID-19 people due to shortage in the material of testing and further increased by the suboptimal personal protective equipment (PPE) available for health personnel in low-and middle-income countries (LMICs) [5]. Surgeons are particularly affected due to the wide variety of procedures they perform, many of which are conducted routinely in the outpatient setting. Urgent measures are warranted to avert health specialist from being infected or dying of COVID-19. This ensures that we have readily available healthy surgeons, anesthesiologists, intensivists and nurses for an adequate perioperative management of COVI-19 patients with surgical pathologies. Considering the important role of surgical teams during the COVID-19 pandemic many scientific societies have set-up guidelines to curb the prevalence of COVID-19 among surgical teams. Some of these recommendations strategies include postponing all elective surgical procedures, triage of patients to identify those at risk of COVID-19, compulsory surgical care of COVID-19 patients surgical emergencies, a constant supply of adequate PPE for the surgical team, wearing of a face mask by the COVID-19 patient during the entire surgical intervention. Designated COVID-19 operating areas (COA) must be allocated for COVID-19 patients requiring an emergency surgery. Patients transit to and from the COA must be as quick as possible [6].

Zheng L and colleagues have also put forth a protocol of emergency surgery based on three level of stratified risks: (i) For confirmed and suspected COVID-19 patients, surgeons need to report to the hospital´s epidemiology department, infection control department, and operating room before they undergo surgery and then transfer a separate path to a negative pressure room, then to an isolation area. (ii) For high-risk COVID-19 patients who have to undergo surgery, after their preoperative preparation is completed, the anesthesiologist, nurse, and surgeon all follow protective measures for anesthesia and surgical procedures. After the surgery, the patients return to the original isolation ward according to the original transfer route. (iii) For low-risk COVID-19 patients, the general protection measures are needed for anesthesia and surgical procedures. After the operation, patients are transferred to their original ward according to the original transfer route [7]. In LLCs, surgical interventions are generally expensive for the general population [8] and the reduction of elective surgery may contribute to inflate this cost. Of note, the setting in which surgical interventions are performed, might be an important risk to acquire COVID-19. Thus, it is urgent to propose some practical strategies and resource-sensitive and context specific guidelines to reduce the spread of the disease in the operating room. Pending to resource availability and constraints, these strategic recommendations could be summarized in nine points as follow: 1) Adequate triage of all patients who undergo surgical intervention by assessing fever or respiratory signs; 2) Addressing all suspected patients to a specific unit for rapid diagnostic testing; 3) Interim cancelation of postponing of all elective surgery for confirmed cases; 4) Multidisciplinary operative staff meeting should be organized by video-communications or webinars; 5) Social distancing during clinical examination ; 6) Patients should wear their masks during all the peri-operative time (pre-operative consultation, during the entire surgical intervention and thereafter in the post-operative recovery unit) ; 7) The surgical team should have a constant and readily supply of PPE; 8) Sterilization of all surgical equipments and the operating room after surgery, especially after operating COVID-19 confirmed case; 9) Restrictions of family and friends visits to the patient in the postoperative period.

A few couples of days succeeding the COVID-19 confinement in some high-income countries the pandemic of higher risk of transmission of COVID-19 increased in the general population as well as several surgical services given that no curative treatment nor vaccine for COVID-19 has been discovered yet. Hence, the surgical workforce or surgical team is continuously being re-inforced through psychological support to prevent physical and emotional exhaustion; enhancing their healthcare leadership and capacity building skills; regular clear communication between surgeons, between surgeons and anesthesiologists and between the surgical team and surgical patients; continuous refresher courses for the surgical teams on PPE; securing enough PPE to protect both the surgical patient and surgical team; continuous compliance to preventive measures such as hand washing before and after surgery; respecting social distancing during the surgical intervention [9]. While emergency surgical procedures will need to continue to be performed using PPE, elective or non-essential surgeries might still remain unfeasible in LMICs due to a shortage of PPE for the surgical human resources [10]. The following checklist proposed by the Royal College of Surgeons in the UK should help in prioritization of surgical interventions and resources adapted to local healthcare system: adequate timing for elective surgery, adequate supply and use of PPE, availability of interdependent service like anesthesiology, critical care or pathology; availability of local testing kits or laboratories for COVID-19, availability of an accurate assessment of the surgical workload by surgical sub-specialties, provision of a minimal risk environment that includes ward areas, operating room and post-anesthesia care units with adequate facilities to care for COVID-19 patients with surgical pathologies as well as to avert infection amongst the surgical team; adequate theatre capacity to meet the surgical service demands; ensuring an adequate supply of a surgical workforce in good physical and mental health; local coordination and availability of recovery management team in place (with multi-professional and multidisciplinary clinical input), to provide coordination and oversight of relevant policies and communications [9]. In poor resource settings, this flexible tool could also be used in order to limit spread of COVID-19 infection in surgical context.

## Conclusion

COVID-19 is a real public health challenge for global healthcare system, impacting severely surgical services. This devastating pandemic calls for urgent readaption and reorientation of health policies and health system to comply with the incident pressing health needs in LMICs. This include implementation of practical context-specific and resources-sensitive guidelines to curb and reduce the transmission rate of SARS-CoV-2 infection to the surgical teams and improve the surgical care of COVID-19 patients living in underserved settings, even during and early after this pandemic.

## Competing interests

All authors declare no competing interests.
